# Composition formulas of Fe-based transition metals-metalloid bulk metallic glasses derived from dual-cluster model of binary eutectics

**DOI:** 10.1038/s41598-017-09100-9

**Published:** 2017-08-22

**Authors:** Gul Jabeen Naz, Dandan Dong, Yaoxiang Geng, Yingmin Wang, Chuang Dong

**Affiliations:** 1 Key Laboratory for Materials Modification by Laser, Ion and Electron Beam (Dalian University of Technology), Ministry of Education, Dalian, 116024 China; 2 Department of Physics, Dalian University, Dalian, 116622 China; 3 School of Materials Science and Engineering, Jiangsu University of Science and Technology, Zhenjiang, 212003 China

## Abstract

It is known that bulk metallic glasses follow simple composition formulas [cluster](glue atom)_1 or 3_ with 24 valence electrons within the framework of the cluster-plus-glue-atom model. Though the relevant nearest-neighbor cluster can be readily identified from a devitrification phase, the glue atoms remains poorly defined. The present work is devoted to understanding the composition rule of Fe-(B,P,C) based multi-component bulk metallic glasses, by introducing a cluster-based eutectic liquid model. This model regards a eutectic liquid to be composed of two stable liquids formulated respectively by cluster formulas for ideal metallic glasses from the two eutectic phases. The dual cluster formulas are first established for binary Fe-(B,C,P) eutectics: [Fe-Fe_14_]B_2_Fe + [B-B_2_Fe_8_]Fe ≈ Fe_83.3_B_16.7_ for eutectic Fe_83_B_17_, [P-Fe_14_]P + [P-Fe_9_]P_2_Fe≈Fe_82.8_P_17.2_ for Fe_83_P_17_, and [C-Fe_6_]Fe_3_ + [C-Fe_9_]C_2_Fe ≈ Fe_82.6_C_17.4_ for Fe_82.7_C_17.3_. The second formulas in these dual-cluster formulas, being respectively relevant to devitrification phases Fe_2_B, Fe_3_P, and Fe_3_C, well explain the compositions of existing Fe-based transition metals-metalloid bulk metallic glasses. These formulas also satisfy the 24-electron rule. The proposition of the composition formulas for good glass formers, directly from known eutectic points, constitutes a new route towards understanding and eventual designing metallic glasses of high glass forming abilities.

## Introduction

Since their first synthesis in 1995, Fe-based bulk metallic glasses (BMGs) have drawn increasing attention due to their soft magnetic properties, high glass forming ability, excellent corrosion resistance, and low manufacturing cost^1–6^. These BMGs are of metal-metalloid type, where the metal concentration is close to 80 at. % and the metalloid elements are mostly P, C, B, and Si^7^. To increase the glass forming ability, multi-element alloying is generally conducted, by adding late transition metals such as Cr and Mo, early transition metals such as Zr, Hf, and Nb, rare earth metals such as Y, La, and trivalent simple metals Al and Ga^8–14^. For instance, the critical rod diameter of fully glassy state can reach 2.5 mm for Fe_77_Ga_3_P_9.5_C_4_B_4_Si_2.5_, 4 mm for Fe_76_Mo_2_Ga_2_P_10_C_4_B_4_Si_2_ and 6 mm for Fe_66_Co_10_Mo_4_(P_0.45_C_0.2_B_0.2_Si_0.15_)_20_^13, 15–17^. To understand and eventually to design these BMGs of such complex chemistry, practical and quantitative alloy design methods should be developed.

In our previous works, the cluster-plus-glue-atom model has been proposed to describe the idealized local units of bulk metallic glasses^18^. The compositions of all binary BMGs can be precisely interpreted^19, 20^. In this model, an amorphous structure of high glass forming ability is dissociated into a characteristic first-neighbor cluster plus one or three glue atoms located between the clusters. In such a definition, there is no solvent or solute. The cluster is derived following stringent procedures from corresponding devitrification crystal. The number of glue atoms, being 1 or 3, comes from an analysis of existing good glass forming compositions^18–20^. These special numbers should be correlated to the way the clusters are packed. As a speculative explanation, if the clusters are densely packed following FCC-like style and if the glue atoms fill the octahedral interstices between the clusters, the number ratio of the cluster and the glue atoms is 1:1, i.e., the number of glue atom is 1. Similarly, the number of 3 might be correlated to a BCC-like inter-cluster configuration, where the glue atoms occupy all the octahedral interstices. The clusters are isolated from each other to avoid the cluster-type local order from being extended to longer-ranges that would eventually break the non-crystalline amorphous state. The total number of valence electrons per unit cluster formula (e/u) is close to 24^21^. In a recent work, standard procedures towards designing BMGs were practiced in Ti-Cu-based BMGs^22^. Two major steps are: (1) analyzing a binary devitrification phase to obtain the principal cluster for use in the relevant glass-forming cluster formula, and (2) matching one or three glue atoms to the principal cluster so that e/u ≈ 24. By principal cluster here we mean the most strongly bonded nearest-neighbor order that is shared between the glassy and the crystalline states. They can be identified from the usually multiple clusters present in a given crystalline phase using high cluster isolation and atomic dense packing criteria^23^. It should be noted that special types of clusters have been clearly identified by computer simulation in Cu-Zr system^24, 25^, and among them, icosahedron Cu_8_Zr_5_ well interprets the Cu_64_Zr_36_ BMG, formulated as [Cu-Cu_7_Zr_5_]Cu~Cu_64.3_Zr_35.7_. However, this method still suffers from a major difficulty in the accurate determination of a cluster formula in the basic binary system, on which multi-alloying is further conducted for enhanced glass forming abilities. While the principal cluster is readily established in a devitrification following the well-established procedures, the appropriate glue atoms are poorly defined, often empirically fitted from known BMG compositions. Also the lack of precise information on cluster radius and atomic density hinders the accurate calculation of e/u. Therefore, despite of our previous success in understanding various metal-metal metallic glass systems such as Cu-(Zr, Hf, Ti), Ni-(Nb, Ta)^19, 20^, the composition rule for metal-metalloid ones is still missing.

In this paper, the cluster-plus-glue-atom structural model and the relevant composition formulas will be used to understand the composition rule of Fe-(B,P,C)-based transition metal-metalloid multicomponent BMGs. The above-mentioned difficulties will be particularly addressed, by introducing a new approach towards obtaining the appropriate Fe-(B,P,C)binary cluster formulas, which is based on the dual cluster formulism for eutectic liquids^26^. After obtaining the basic Fe-(B,P,C) formulas, compositions of some typical Fe-(B,P,C) based binary, ternary and multi-component BMGs will be explored on the basis of the binary basic formulas. It will be shown that the good glass-forming compositions are well interpreted using the cluster formulas containing nearly 24 valence electrons.

## Dual-cluster formulas for eutectic liquids

In this section, the cluster-plus-glue-atom approach for eutectic composition interpretation will be explored in detail, covering 1) dual-cluster model of eutectic liquids, and 2) its application in the composition interpretation of binary Fe-rich eutectic points, close to which most Fe-based BMGs are formed.

### Dual-cluster model of eutectic liquids

As already stated in ref. 26, binary eutectic liquids are characterized, in terms of the cluster-plus-glue-atom model for describing short-range-order local structures, by:

1. *Two stable liquid subunits issued from the two eutectic phases*, *and*
2. *Each subunit formulated as* [*cluster*](*glue atoms*)_*1 or 3*_ *of ideal metallic glasses*_._

A binary eutectic composition is therefore expressed as [cluster_α_ + cluster_β_](glue atoms)_2 or 4 or 6_, where the two clusters in the brackets belongs to the two liquid subunits resulted from the corresponding eutectic phases α and β.

The identification of the right clusters from eutectic phases then becomes the key step toward establishing the dual-cluster model for a eutectic liquid. Here the clusters are derived from the eutectic phases that bound the eutectic point. Yet, in a given crystal structure, there are often multiple nearest-neighbor clusters. There are two criteria for the selection of the right cluster (called the principal cluster) in the cluster formula, the maximum cluster isolation and atomic dense packing^21^. A cluster of such a type satisfies ideal atomic interaction and constitutes the most strongly bonded part in the structure. This cluster is then assumed to be inherited from the liquid state, via the amorphous solid state, down to the devitrification/eutectic phase from which it is derived. The well-measured eutectic points serve a good check for the appropriate matching of glue atoms. In the next part, the Fe-(B,P,C) binary eutectic compositions relevant to Fe-based BMGs will be addressed using the dual-cluster formulism, and from which the basic formulas for glass-forming formulas will be derived.

### Dual-cluster formulas of Fe-rich Fe-(B, P, C) binary eutectics

It has been well-established that BMG compositions satisfy simple composition formulas [principal cluster](glue atom)_1 or 3_ with e/u = 24, where the principal cluster is derived from a devitrification phase. The binary Fe-(B, P, C) BMG-relevant eutectic points will be analyzed via the dual cluster formulism.

For the Fe-B-based BMGs, the relevant eutectic point is Fe_83_B_17_ and the eutectic phases are γ-Fe (FCC, Cu type) and BFe_2_ (tetragonal, Al_2_Cu type).

**Figure 1:**
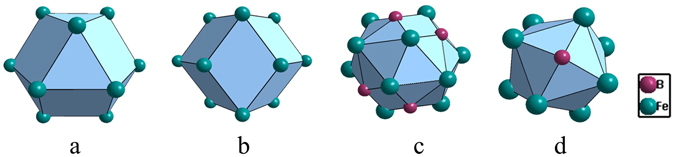
Cuboctahedron CN12 [Fe-Fe_12_] in FCC γ-Fe (**a**), rhombidodecahedron CN14 in [Fe-Fe_14_] BCC δ-Fe(**b**), and CN15 [Fe-B_4_Fe_11_] (**c**) and CN10 octahedral antiprism [B-B_2_Fe_8_] (**d**) in BFe_2_.

The γ-Fe phase is characterized by a unique cuboctahedral cluster [Fe-Fe_12_] (Fig. 1a), typical for FCC metals that do not contain any solute (B is almost insoluble with Fe). In expressing a cluster, the center atom is placed first and is separated from the 1^st^-neighbor ones by a hyphen, both square-bracketed. However, as far as the liquid structure is concerned, on which our model is really based, it is widely accepted that the nearly pure Fe liquid structure is in fact related to the high-temperature BCC *δ*-Fe phase^27^, characterized by a unique rhombidodecahedron [Fe-Fe_14_] cluster (Fig. 1b). Therefore, in dealing with the Fe-B system, the BCC [Fe-Fe_14_] cluster will be use instead.

The other crystalline phase BFe_2_ is the devitrification phase for the Fe-B-based BMGs, and the cluster formula issued from this phase should be responsible for the glass formation. In the unit cell of BFe_2_ (Al_2_Cu structure type), there are two non-equivalent sites, B at (0, 0, 0.25) and Fe at (0.1661, 0.6661, 0). All crystal structure data are taken from Pearson’s handbook^28^. Two clusters can be defined centered by the two sites, i.e., Fe-centered [Fe-B_4_Fe_11_] with coordination number (CN) of 15 (Fig. 1c), and a B-centered Archimedean octahedral antiprism CN10 [B-B_2_Fe_8_] (Fig. 1d).

**Figure 2:**
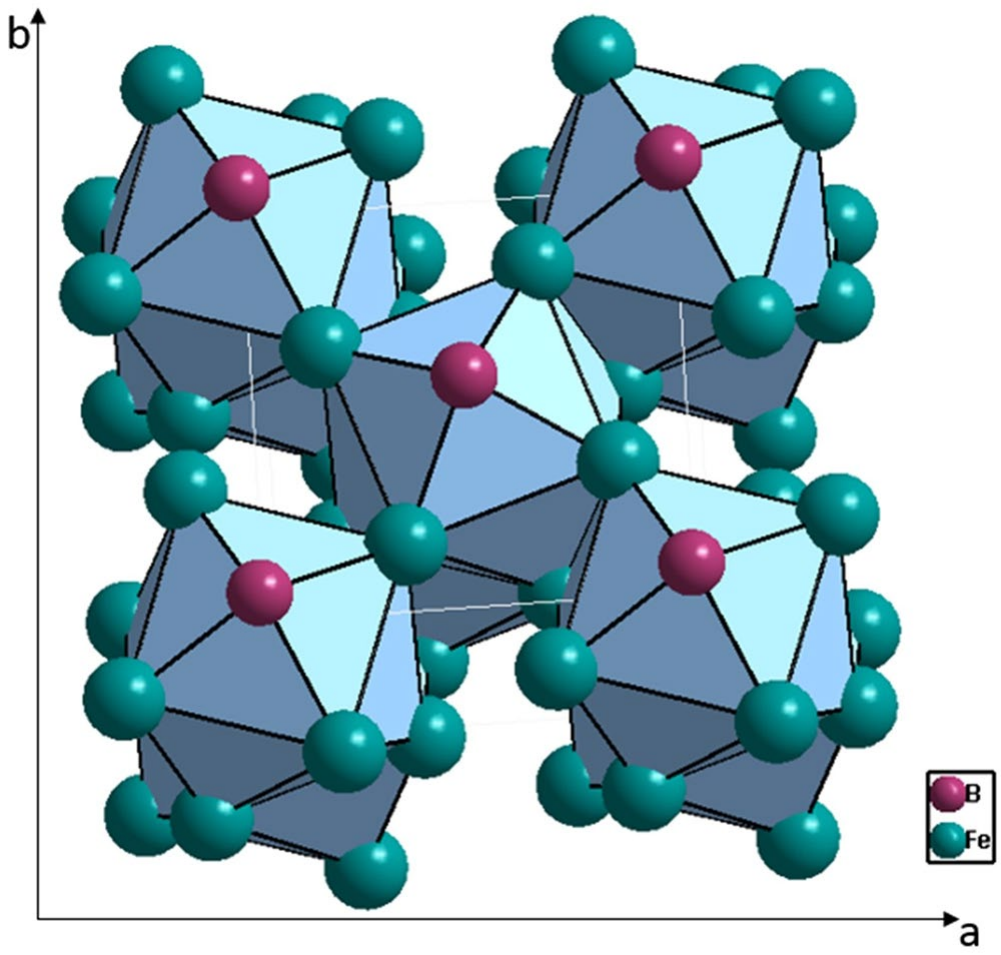
Inter-connection of B-centered [B-B_2_Fe_8_] clusters in BFe_2_ showing extensive overlapping.

The degree of cluster isolation is measured by comparing the complete cluster size (i.e., the number of atoms in the cluster) with the one with a reduced size after considering inter-cluster overlapping. For the ideal case in which no overlap between the neighboring clusters occurs, the two sizes are equal. However, in general, clusters overlap with each other due to the periodic constraint. The two clusters [Fe-B_4_Fe_11_] and [B-B_2_Fe_8_] are respectively reduced to [Fe-B_0.5_] and [B-Fe_2_] (Fig. 2) after overlapping, with the latter demonstrating a better cluster isolation (a larger reduced cluster size of three atoms). Therefore, the B-centered [B-B_2_Fe_8_] cluster is selected as the principal cluster to enter into the BFe_2_-relevant glass-forming formula.

**Figure 3:**
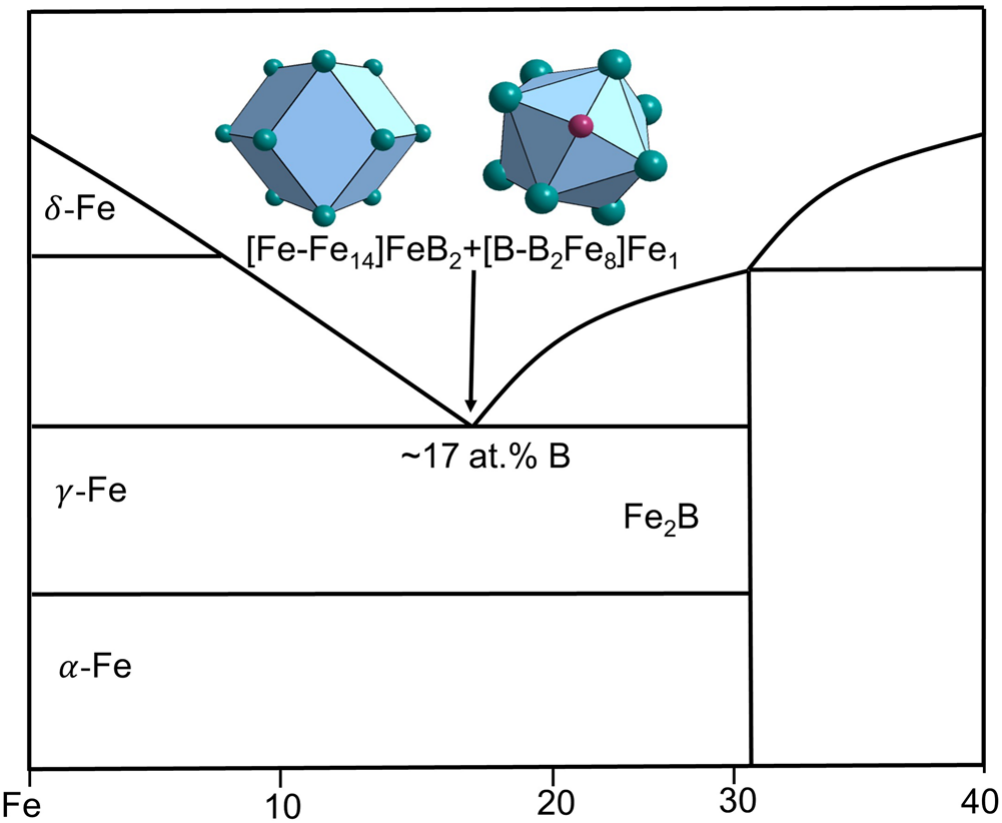
Interpretation of Fe_83_B_17_ eutectic point using a dual-cluster formula [Fe-Fe_14_]FeB_2_ + [B-B_2_Fe_8_]Fe_1_, with the two clusters being derived respectively from eutectic phases δ-Fe (W type) and BFe_2_ (AlCu_2_).

Then, two principal clusters [Fe-Fe_14_] and [B-B_2_Fe_8_] are respectively derived. After trying all combinations of two, four, and six glue atoms of Fe and B, the two clusters plus four glue atoms B_2_Fe_2_ matches perfectly the experimental eutectic point at Fe_83_B_17_: [Fe-Fe_14_ + B-B_2_Fe_8_]B_2_Fe_2_ = Fe_25_B_5_ ≈ Fe_83.3_B_16.7_ (Fig. 3).

Next step is to assign the four glue atoms to each cluster, producing two individual cluster formulas. There are actually only two kinds of combinations for glue atoms, B_2_Fe-Fe and BFe_2_-B. [B-B_2_Fe_8_], after being matched with each of the four possibilities, produces the following formulas: [B-B_2_Fe_8_]Fe = Fe_75_B_25_, [B-B_2_Fe_8_]BFe_2_ = Fe_71.4_B_28.6_, [B-B_2_Fe_8_]B = Fe_66.7_B_33.3_, and [B-B_2_Fe_8_]B_2_Fe = Fe_64.3_B_35.7_. Melt-quenched Fe-B metallic glasses are usually formed in the composition range of 72–88 at.% Fe^29–32^. The Fe-richest [B-B_2_Fe_8_]Fe is then chosen because it is the only one falling in the glass forming range. Recently, we have shown that the optimal glass forming composition is exactly at the composition defined by the established formula [B-B_2_Fe_8_]Fe = Fe_75_B_25_^33^. It will also be illustrated that this formula is responsible for most Fe-B-based BMGs. The determination of cluster formula for glass formation from dual cluster formulism of eutectic liquid therefore constitutes a simple and accurate route towards BMG formulation, which overcomes the ambiguity in glue atom definition.

The Fe-P based BMGs are related to eutectic point Fe_83_P_17_, involving two eutectic phases *α*-Fe and Fe_3_P.

α-Fe (BCC, W type) is characterized by the same rhombidodecahedral cluster as *δ*-Fe (Fig. 1b). However, considering the substantial solubility of P in α-Fe and the negative enthalpy of mixing between P and Fe, it is reasonable to assume a P-centered [P-Fe_14_] cluster, rather than [Fe-Fe_14_] as in the Fe-B case.

**Figure 4:**
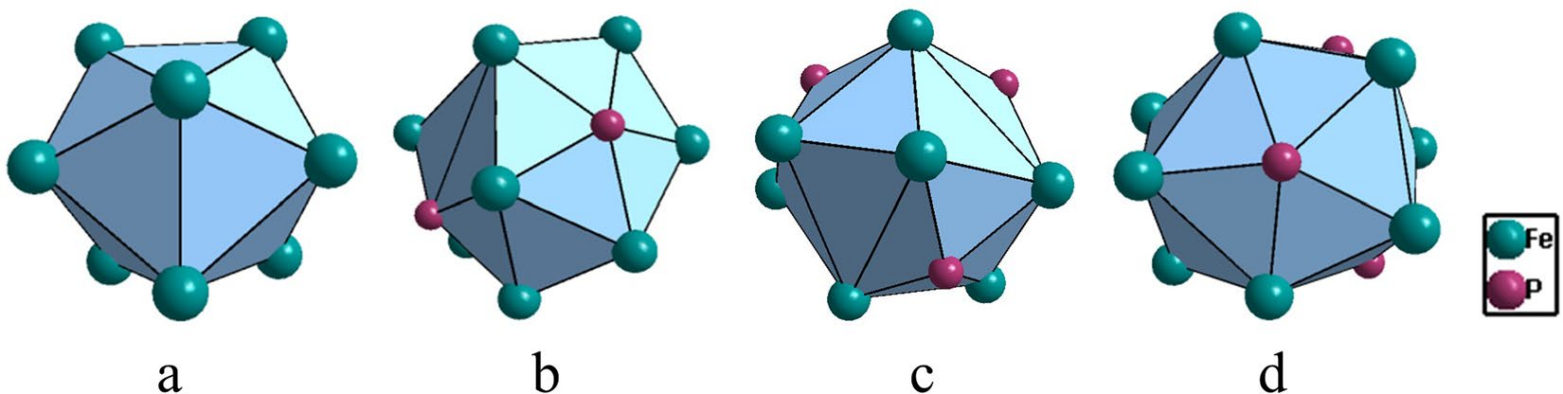
Clusters in Fe_3_P: capped trigonal prism [P-Fe_9_] (**a**), CN14 [Fe-P_2_Fe_12_] (**b**), CN13 [Fe-P_3_Fe_10_] (**c**), and CN14 [Fe-P_4_Fe_10_] (**d**).

Fe_3_P is a commonly identified devitrification phase for BMGs^34, 35^. It is of Ni_3_P structure type. There exist four non-equivalent sites in its unit cell, one being occupied by P and the other three by Fe’s. From the four sites are developed four clusters, i.e., capped trigonal prism CN9 [P-Fe_9_], CN14 [Fe-P_2_Fe_12_], CN13 [Fe-P_3_Fe_10_] and CN14 [Fe-P_4_Fe_10_] (Fig. 4). The degree of isolation is the highest for the P-centered one, with the reduced cluster being [P-Fe_3_]. The Fe-centered clusters are reduced to [Fe-PFe_2_], but from much larger initial sizes than the P-centered one. Therefore, the P-centered capped trigonal prism [P-Fe_9_] is taken as the principal cluster, instead of any of the Fe-centered ones.

**Figure 5:**
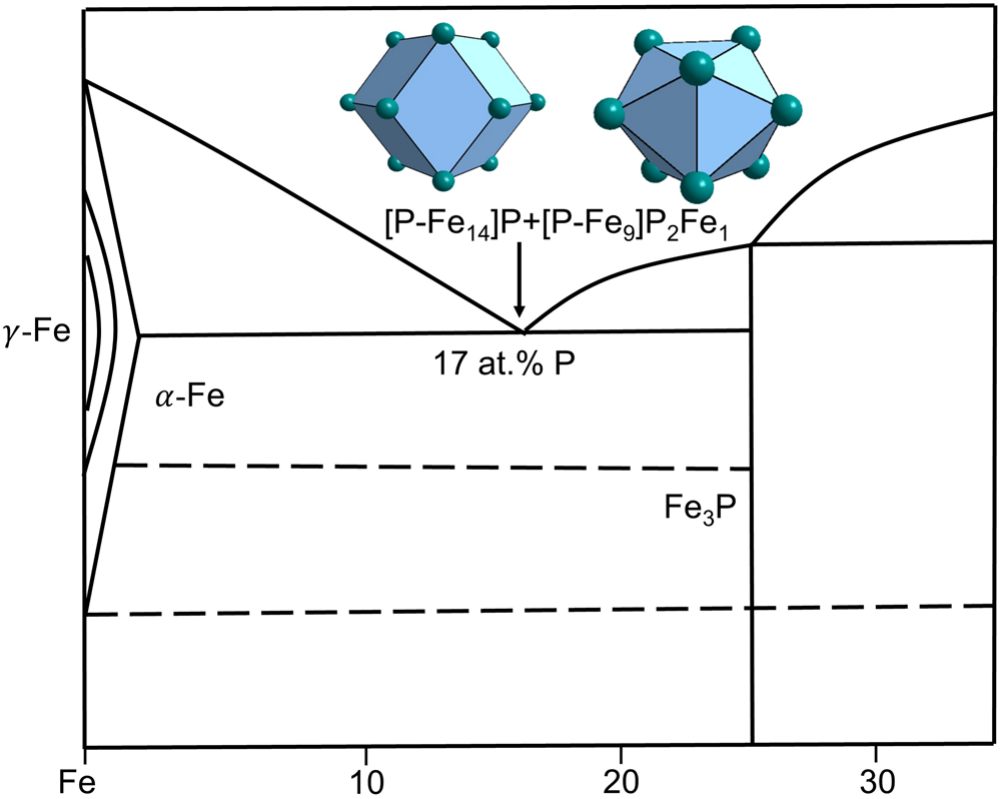
Interpretation of eutectic point Fe_83_P_17_ by the dual-cluster formula [P-Fe_14_]P + [P-Fe_9_]P_2_Fe derived from eutectic phases α-Fe and Fe_3_P (Ni_3_P).

After matching with appropriate glue atoms, a dual cluster formula is proposed to interpret the eutectic point composition Fe_83_P_17_: [P-Fe_14_ + P-Fe_9_]P_3_Fe = Fe_24_P_5_ ≈ Fe_82.8_P_17.2_ (Fig. 5).

There are two options to separate the glue atoms P_3_Fe, either P_3_-Fe or P_2_Fe-P_1_. [P-Fe_9_], after being matched with each of the four possibilities, gives [P-Fe_9_]Fe = Fe_90.9_P_9.1_, [P-Fe_9_]P_1_ = Fe_81.8_P_18.2_, [P-Fe_9_]P_2_Fe = Fe_76.9_P_23.1_, and [P-Fe_9_]P_3_ = Fe_69.2_P_30.8_. Fe-P metallic glasses have been obtained by liquid quenching over a composition range of 13~24 at.% P^36, 37^. Among the four formulas, [P-Fe_9_]P_2_Fe is chosen as it is in the glass forming zone. [P-Fe_9_]P_1_, being also in the range but quite close to the eutectic point, is eliminated because glass formation composition usually deviates from the eutectic one^38^. As will be illustrated later, [P-Fe_9_]P_2_Fe is indeed responsible for BMG formation in multi-component Fe-P-based alloys.

**Figure 6:**
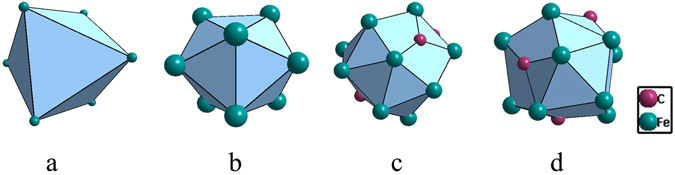
Clusters in C-containing γ-Fe (**a**: octahedron) and in CFe_3_ (**b**) capped trigonal prism CN9 [C-Fe_9_]), (**c**) CN15 [Fe-C_3_Fe_12_], and (**d**) CN 14 [Fe-C_3_Fe_11_].

Fe-C-based BMGs are related to eutectic point Fe_83_C_17_, involving two eutectic phases *γ*-Fe and Fe_3_C. *γ*-Fe (FCC, Cu type) is characterized by cuboctahedral cluster [Fe-Fe_12_]; however, since it dissolves a substantial amount of C in its octahedral interstitial site, the more reasonable cluster should be C-centered octahedron [C-Fe_6_] (Fig. 6a), rather than [Fe-Fe_12_] (when a substitutional type of solute is nearly insoluble) or [C-Fe_12_] (C is too small to be a substitutional element).

The commonly observed devitrification phase for the Fe-C-based BMGs is cementite Fe_3_C. The three non-equivalent sites, one C and two Fe, define capped trigonal prism CN9 [C-Fe_9_], CN15 [Fe-C_3_Fe_12_], and CN 14 [Fe-C_3_Fe_11_] (Fig. 6). These clusters are reduced respectively to [C-Fe_3_], [Fe-CFe_2_], and [Fe-C_0.5_Fe_0.5_]. Therefore, the first one [C-Fe_9_] is chosen as the principal cluster for Fe_3_C, which is the same cluster type as [P-Fe_9_] from Fe_3_P.

**Figure 7:**
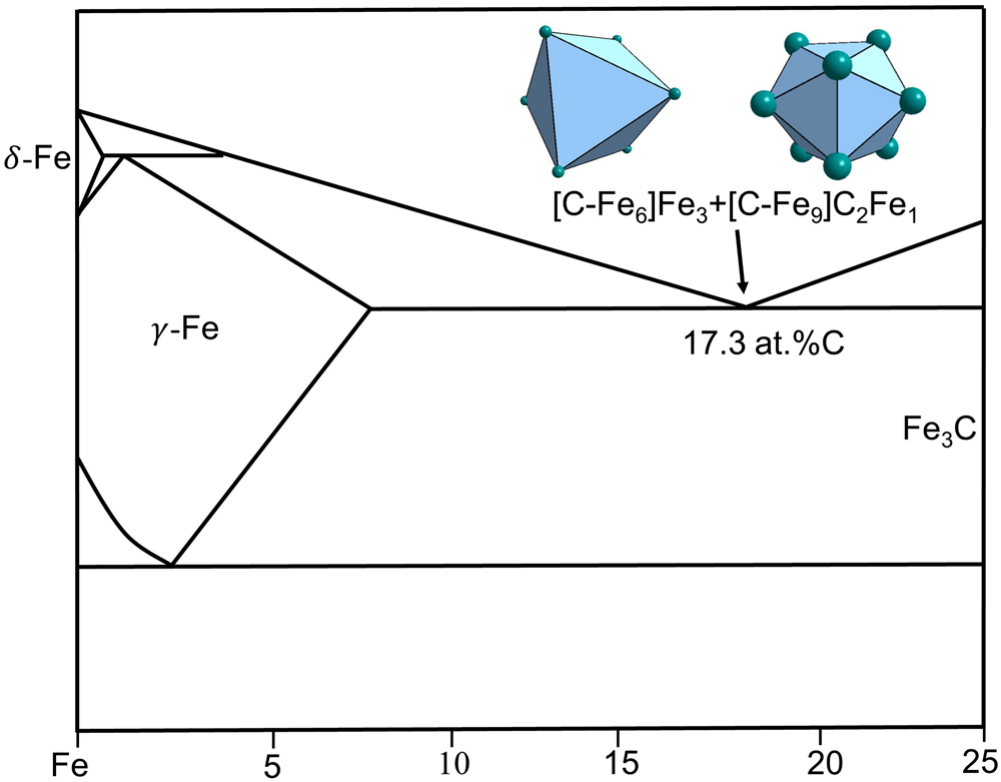
Interpretation of Fe_82.7_C_17.3_ eutectic point by the dual-cluster formula [C-Fe_6_]Fe_3_ + [C-Fe_9_]C_2_Fe from eutectic phases γ-Fe(C) and Fe_3_C.

The eutectic point Fe_82.7_C_17.3_ is explained by the dual cluster formula: [C-Fe_6_ + C-Fe_9_]Fe_4_C_2_ = Fe_19_C_4_ ≈ Fe_82.6_C_17.4_. There are two possibilities to separate the Fe_4_C_2_ glue atoms: Fe_3_-Fe_1_C_2_ and Fe_2_C_1_-Fe_2_C_1_. The [C-Fe_9_] cluster, after being matched with each of the three possibilities, produces cluster formulas [C-Fe_9_]Fe_3_ = Fe_92.3_C_7.7_, [C-Fe_9_]Fe_2_C_1_ = Fe_84.6_C_15.4_, and [C-Fe_9_]Fe_1_C_2_ = Fe_76.9_C_23.1_. The only reasonable formula is the last one because the first and the second ones fall on the other side of the eutectic point. Therefore, the only possible pair of cluster formulas, as shown in Fig. 7, are [C-Fe_6_]Fe_3_ + [C-Fe_9_]C_2_Fe. The latter formula is expected to correspond to good glass formers because it is related to a devitrification phase. For Fe-C binary alloys, the glass forming ability is weak and there is no report on the best glass former in this system, though the glassy state by liquid quenching has been reported near eutectic point^35^.

From the dual cluster formulas for binary eutectics, three basic cluster formulas are established: [B-B_2_Fe_8_]Fe, [P-Fe_9_]P_2_Fe and [C-Fe_9_]C_2_Fe, which are related to devitrification phases Fe_2_B, Fe_3_P, and Fe_3_C, respectively. They will be used as the basis for BMG composition analysis.

## Cluster formulas for Fe-based metallic glasses

Fe-based metallic glasses usually have complex compositions but can always be regarded as being developed from M-(B,C,P) binary systems^14^.

Existing Fe-(B,C,P)-based multi-component compositions with good glass forming abilities are carefully scrutinized using the proposed binary basic formulas within the framework of the cluster-plus-glue atom model. The Fe-(B,C,P)-based multi-component compositions are shown in Table 1. As the example, BMG Fe_75_Mo_4_B_4_C_4_Si_3_P_10_ of a critical size of 4 mm is chosen to illustrate the procedures of composition interpretation, as stated below.

1. Select the alloys with good glass forming abilities;
  This step guarantees, to the maximum degree, that the compositions selected corresponds to high glass forming ability, as our approach only works for ideal metallic glasses stabilized at specific compositions. This is of course often inaccurate, especially in multi-component systems, as the trial-and-error experiments become quite tedious. In BMG series Fe_79−*x*_Mo_*x*_P_10_C_4_B_4_Si_3_ (*x* = 0–6 at. %), the composition with the maximum diameter thickness is Fe_75_Mo_4_B_4_C_4_Si_3_P_10_^77^.
2. Classify the elements into transition metals (M), Rare Earth metals (RE), simple metals Al and Ga, and metalloids;
  Fe_75_Mo_4_B_4_C_4_Si_3_P_10_ is then converted into (Fe_75_Mo_4_)(B_4_C_4_Si_3_P_10_) = M_79_(B_4_C_4_Si_3_P_10_), where M = (Fe_0.95_Mo_0.05_).
3. Determine the basic binary formula for glass formation;
  Being P-rich, M_79_(B_4_C_4_Si_3_P_10_) should be based on the Fe-P binary formula [P-Fe_9_]P_2_Fe.
4. Convert the atomic percent composition into the cluster formula;
  By multiplying the total number of atoms per unit cluster formula (*Z*), the atomic percent composition is converted to the cluster formula of the alloy following the basic binary formulas. The basic formula containing *Z* = 13 atoms, the composition Fe_75_Mo_4_B_4_C_4_Si_3_P_10_ = M_79_(B_4_C_4_Si_3_P_10_) is multiplied by 13 to obtain Fe_9.8_Mo_0.5_B_0.5_C_0.5_Si_0.4_P_1.3_ = M_10.3_B_0.5_C_0.5_Si_0.4_P_1.3_ = [P-M_9_]M_1.3_B_0.5_C_0.5_Si_0.4_P_0.3_ M = (Fe_0.95_Mo_0.05_).
5. Confirm the 24-electron rule;

**Table 1:** Fe-metalloid-based bulk metallic glass compositions and their interpretation using the binary cluster formulas [B-B2Fe8]Fe1 (Z = 12, r1 = 0.2170 nm), [P-Fe9]P2Fe (Z = 13, r1 = 0.2337 nm), and [C-Fe9]C2Fe (Z = 13, r1 = 0.2184 nm).

Compositions, at. %	Average transition metals M	Cluster formulas	Critical size/mm	Mass density ρ/g.cm^−3^		e/u	Ref.
				Cal.	Exp.		
**[B**-**B**_**2**_ **Fe**_**8**_]**Fe**_**1**_-**based**							
Fe_75_Si_10_B_15_ = M_75_Si_10_B_15_	Fe	[B-B_0.8_Si_1.2_M_8_]M_1_	40 µ	7.19	7.14	25.8	86
Fe_73.8_Cr_8.2_B_18_ = M_82_B_18_	Fe_0.9_Cr_0.1_	[B-B_1.2_M_8.8_]M_1_	—	7.4	—	25.7	40
Fe_73_V_9_B_18_ = M_82_B_18_	Fe_0.89_V_0.11_	[B-B_1.2_M_8.9_]M_1_	—	7.2	—	26.2	40
Fe_40_Ni_40_B_20_ = M_80_B_20_	Fe_0.5_Ni_0.5_	[B-B_1.4_M_8.6_]M_1_	—	7.8	—	24.7	40, 41
Fe_72_Y_6_B_22_ = M_72_Y_6_B_22_	Fe	[B-B_1.6_Y_0.7_M_7.6_]M_1_	2	6.8	—	28.1	32, 42
Fe_72_Sc_6_B_22_ = M_72_Sc_6_B_22_	Fe	[B-B_1.6_Sc_0.7_M_7.6_]M_1_	2	6.7	—	26.8	42
Fe_72_Er_6_B_22_ = M_72_Er_6_B_22_	Fe	[B-B_1.6_Er_0.7_M_7.6_]M_1_	2	7.6	—	27.7	42
Fe_69_Co_16_B_15_ = M_85_B_15_	Fe_0.81_Co_0.19_	[B-B_0.8_M_9.2_]M_1_	—	7.6	—	26.1	43
Fe_69.7_Ga_15.3_B_15_ = M_69.7_Ga_15.3_B_15_	Fe	[B-B_0.8_Ga_1.8_M_7.4_]M_1_	—	7.8	—	26.2	44
Fe_70_Zr_10_B_20_ = M_70_Zr_10_B_20_	Fe	[B-B_1.4_Zr_1.2_M_7.4_]M_1_	—	7.1	—	28.2	45, 46
Fe_66_Nb_4_B_30_ = M_70_B_30_	Fe_0.94_Nb_0.06_	[B-B_2.6_M_7.4_]M_1_	1	7.4	—	23.8	47
Fe_65_Nb_3_B_32_ = M_68_B_32_	Fe_0.96_Nb_0.04_	[B-B_2.8_M_7.2_]M_1_	—	7.3	—	23.2	47
Fe_65.7_Nb_3.5_B_30.8 = _M_69.2_B_30.8_	Fe_0.95_Nb_0.05_	[B-B_2.7_M_7.3_]M_1_	—	7.3	—	23.5	47
Fe_68_Nb_4_B_28_ = M_72_B_28_	Fe_0.94_Nb_0.06_	[B-B_2.4_M_7.6_]M_1_	—	7.4	—	24.2	47
Fe_71_Nb_6_B_23_ = M_77_B_23_	Fe_0.92_Nb_0.08_	[B-B_1.8_M_8.2_]M_1_	1.5	7.4	7.3	26.1	72
Fe_71.2_Y_4.8_B_24_ = M_71.2_Y_4.8_B_24_	Fe	[B-B_1.9_Y_0.6_M_7.5_]M_1_	1	6.8	6.8	27.3	48
Fe_76_B_10_Si_9_P_5_ = M_76_B_10_Si_9_P_5_	Fe	[B-B_0.2_Si_1.1_P_0.6_M_8.1_]M_1_	2.5	6.9	7.2	26.4	49
Fe_30_Co_30_Ni_15_Si_8_B_17_ = M_75_B_17_Si_8_	Fe_0.4_Co_0.4_Ni_0.2_	[B-B_1_Si_1_M_8_]M_1_	1.2	7.6	—	24.7	49
Fe_72_Nb_4_B_14.4_Si_9.6_ = M_76_B_14.4_Si_9.6_	Fe_0.95_Nb_0.05_	[B-B_0.7_Si_1.2_M_8.1_]M_1_	1.5	7.3	7.3	26.3	50
Fe_72_Nb_4_B_19.2_Si_4.8_ = M_76_B_19.2_Si_4.8_	Fe_0.95_Nb_0.05_	[B-B_1.3_Si_0.6_M_8.1_]M_1_	2	7.3	7.3	25.9	51
Fe_43.2_Nb_4_Ni_28.8_B_19.2_Si_4.8_ = M_76_B_19.2_Si_4.8_	Fe_0.57_Nb_0.05_Ni_0.38_	[B-B_1.3_Si_0.6_M_8.1_]M_1_	2	7.6	—	25.4	51
Fe_36_Nb_4_Co_36_B_19.2_Si_4.8_ = M_76_B_19.2_Si_4.8_	Fe_0.47_Nb_0.05_Co_0.47_	[B-B_1.3_Si_0.6_M_8.1_]M_1_	5	7.6	—	25.2	52
Fe_43.2_Nb_4_Co_21.6_Ni_7.2_B_19.2_Si_4.8_ = M_76_B_19.2_Si_4.8_	Fe_0.57_Nb_0.05_Co_0.28_Ni_0.1_	[B-B_1.3_Si_0.6_M_8.1_]M_1_	4	7.6	—	25.3	52
Fe_72_Nb_4_B_20_Si_4_ = M_76_B_20_Si_4_	Fe_0.95_Nb_0.05_	[B-B_1.4_Si_0.5_M_8.1_]M_1_	2	7.3	7.3	25.8	53, 54
**[P**-**Fe**_**9**_]**P**_**2**_ **Fe**-**based**							
Fe_40_Ni_40_P_20_ = M_80_P_20_	Fe_0.5_Ni_0.5_	[P-M_9_]P_1.6_M_1.4_	—	8.6	—	24.4	55
Fe_78_P_13_C_9_ = M_78_P_13_C_9_	Fe	[P-M_9_](C_1.2_P_0.7_)M_1.1_	2	7.1	7.32	23.6	34
Fe_80_P_13_C_7_ = M_80_P_13_C_7_	Fe	[P-M_9_](C_0.9_P_0.7_)M_1.4_	2	7.1	7.35	24.1	56–61
Fe_41.5_Ni_41.5_P_17_ = M_83_P_17_	Fe_0.5_Ni_0.5_	[P-M_9_]P_1.2_M_1.8_	15 µ	8.5	—	24.6	62
Fe_80_P_13_B_7_ = M_80_P_13_B_7_	Fe	[P-M_9_](B_0.9_P_0.7_)M_1.4_	—	7.1	—	24.1	63
Fe_80_P_11_C_9_ = M_80_P_11_C_9_	Fe	[P-M_9_](C_1.2_P_0.4_)M_1.4_	1.5	6.8	—	23.8	64
Fe_70.55_Ni_12.45_P_17_ = M_83_P_17_	Fe_0.85_Ni_0.15_	[P-M_9_]P_1.2_M_1.8_	—	8.2	—	25.0	65
Fe_70.55_Cr_12.45_P_17_ = M_83_P_17_	Fe_0.85_Cr_0.15_	[P-M_9_]P_1.2_M_1.8_	—	7.9	—	25.2	65
Fe_70.55_Mo_12.45_P_17_ = M_83_P_17_	Fe_0.85_Mo_0.15_	[P-M_9_]P_1.2_M_1.8_	—	8.4	—	26.3	65
Fe_44_Pd_36_P_20_ = M_80_P_20_	Fe_0.55_Pd_0.45_	[P-M_9_]P_1.6_M_1.4_	—	9.9	—	27.6	66
Fe_45_Mn_35_B_7_C_3_P_10_ = M_80_B_7_C_3_ P_10_	Fe_0.56_Mn_0.44_	[P-M_9_](B_0.9_C_0.4_P_0.3_)M_1.4_	2	7.1	7.071	23.8	87
Fe_65_Co_10_Ga_5_B_4_C_4_P_12_ = M_75_Ga_5_B_4_C_4_P_12_	Fe_0.87_Co_0.13_	[P-M_9_](Al_0.7_B_0.5_C_0.5_P_0.6_)M_0.7_	4	7.3	7.3	23.8	67, 68
Fe_80_B_4_C_5_P_11_ = M_80_B_4_C_5_P_11_	Fe	[P-M_9_](B_0.5_C_0.7_P_0.4_)M_1.4_	—	7.1	—	23.8	14
Fe_73_Al_5_Ga_2_B_4_C_5_P_11_ = M_73_Al_5_Ga_2_B_4_C_5_P_11_	Fe	[P-M_9_](Al_0.6_Ga_0.3_B_0.5_C_0.7_P_0.4_)M_0.5_	1.5	6.9	—	23.8	14, 69
Fe_72_Al_5_Ga_2_B_4_C_6_Si_1_P_10_ = M_72_Al_5_Ga_2_B_4_C_6_Si_1_P_10_	Fe	[P-M_9_](Al_0.6_Ga_0.3_B_0.5_C_0.8_Si_0.1_P_0.3_)M_0.4_	2	6.9	—	23.7	70
Fe_75_Ga_5_B_4_C_4_P_12_ = M_75_Ga_5_B_4_C_4_P_12_	Fe	[P-M_9_](Ga_0.7_B_0.5_C_0.5_P_0.6_)M_0.7_	2.5	7.2	—	23.9	71
Fe_77_Ga_3_B_4_C_4_Si_2.5_P_9.5_ = M_77_Ga_3_B_4_C_4_Si_2.5_P_9.5_	Fe	[P-M_9_](Ga_0.4_B_0.5_C_0.5_Si_0.3_P_0.2_)M_1_	2.5	7.1	—	24.1	72, 73
Fe_75_Mo_2_Ga_3_B_4_C_4_Si_2_P_10_ = M_77_Ga_3_B_4_C_4_Si_2_P_10_	Fe_0.97_Mo_0.03_	[P-M_9_](Ga_0.4_B_0.5_C_0.5_Si_0.3_P_0.3_)M_1_	2.5	7.2	—	24.2	35
Fe_62_Co_5_Cr_4_Mo_4_Ga_4_B_5_C_5_P_11_ = M_75_Ga_4_B_5_C_5_P_11_	Fe_0.83_Co_0.07_Cr_0.05_Mo_0.05_	[P-M_9_](Ga_0.5_B_0.7_C_0.7_P_0.4_)M_0.7_	3	7.3	—	23.9	74
Fe_71_Mo_5_B_2_C_10_P_12_ = M_76_ B_2_C_10_P_12_	Fe_0.93_Mo_0.07_	[P-M_9_](B_0.3_C_1.3_P_0.6_)M_0.8_	3	7.3	—	23.6	75
Fe_65_Cr_2_Mo_9_B_6_C_8_P_10_ = M_76_B_6_C_8_P_10_	Fe_0.85_Cr_0.03_Mo_0.12_	[P-M_9_](B_0.8_C_1_P_0.3_)M_0.9_	2.5	7.3	—	23.8	75
Fe_74_Mo_6_B_2.5_C_7.5_P_10_ = M_80_B_2.5_C_7.5_P_10_	Fe_0.92_Mo_0.08_	[P-M_9_](B_0.3_C_1_P_0.3_)M_1.4_	3	7.3	—	24.3	76
Fe_79_B_4_C_4_Si_3_P_10_ = M_79_B_4_C_4_Si_3_P_10_	Fe	[P-M_9_](B_0.5_C_0.5_Si_0.4_P_0.3_)M_1.3_	1	7.1	—	24.0	77
Fe_75_Mo_4_B_4_C_4_Si_3_P_10_ = M_79_B_4_C_4_Si_3_P_10_	Fe_0.95_Mo_0.05_	[P-M_9_](B_0.5_C_0.5_Si_0.4_P_0.3_)M_1.3_	4	7.2	—	24.4	77
Fe_62.9_Ni_11.1_Mo_6_B_2.5_C_7.5_P_10_ = M_80_B_2.5_C_7.5_P_10_	Fe_0.79_Ni_0.14_Mo_0.07_	[P-M_9_](B_0.3_C_1_P_0.3_)M_1.4_	3	7.4	—	24.1	76
**[C**-**Fe**_**9**_]**C**_**2**_ **Fe**-**based**							
Fe_65_Mo_14_B_6_C_15_ = M_79_B_6_C_15_	Fe_0.82_Mo_0.18_	[C-M_9_](B_0.8_C_0.9_)M_1.3_	4	8.7	—	25.3	78, 79
Fe_57.8_Co_6.4_Mo_14_C_15_B_6_Er_0.75_ = M_78.25_Er_0.75_ B_6_C_15_	Fe_0.74_Co_0.08_Mo_0.18_	[C-M_9_]Er_0.1_(B_0.8_C_1_)M_1.1_	4	8.8	—	25.5	78
Fe_57.6_Co_6.4_Mo_14_C_15_B_6_Er_1_ = M_78_Er_1_B_6_C_15_	Fe_0.74_Co_0.08_Mo_0.18_	[C-M_9_]Er_0.1_(B_0.8_C_1)_M_1.1_	3.5	8.8	—	25.6	78
Fe_61_Cr_4_Mo_14_B_6_C_15_ = M_79_B_6_C_15_	Fe_0.77_Cr_0.05_Mo_0.18_	[C-M_9_](B_0.8_C_0.9_)M_1.3_	2	8.7	—	25.3	78
Fe_48_Cr_15_Mo_14_B_6_C_15_Er_2_ = M_77_Er_2_B_6_C_15_	Fe_0.62_Cr_0.2_Mo_0.18_	[C-M_9_]Er_0.3_(B_0.8_C_0.9_)M_1.0_	12	8.7	—	26.3	11
Fe_49_Cr_15_Mo_14_B_6_C_15_Er_1_ = M_78_Er_1_B_6_C_15_	Fe_0.62_Cr_0.2_Mo_0.18_	[C-M_9_]Er_0.1_(B_0.8_C_0.9_)M_1.1_	6	8.6	—	25.8	80
Fe_49_Cr_15_Mo_14_B_4_C_17_Er_1_ = M_78_Er_1_B_4_C_17_	Fe_0.62_Cr_0.2_Mo_0.18_	[C-M_9_]Er_0.1_(B_0.5_C_1.2_)M_1.1_	4	8.6	—	25.8	80
Fe_52.6_Co_5.9_Cr_6_Mo_14_C_15_B_6_Er_0.5_ = M_78.5_Er_0.5_B_6_C_15_	Fe_0.67_Cr_0.08_Mo_0.18_Co_0.07_	[C-M_9_]Er_0.1_(B_0.8_C_0.9_)M_1.2_	4	8.8	—	25.4	80
Fe_50_Cr_15_Mo_14_B_6_C_15_ = M_79_B_6_C_15_	Fe_0.63_Cr_0.19_Mo_0.18_	[C-M_9_](B_0.8_C_0.9_)M_1.2_	1.5	8.6	—	25.4	11
Fe_52_Cr_15_Mo_9_B_6_C_15_Er_3_ = M_76_Er_3_B_6_C_15_	Fe_0.68_Cr_0.2_Mo_0.12_	[C-M_9_]Er_0.4_(B_0.8_C_0.9_)M_0.9_	6	8.5	—	26.3	11
Fe_48_Cr_15_Mo_14_B_6_C_15_Y_2_ = M_77_Y_2_B_6_C_15_	Fe_0.62_Cr_0.2_Mo_0.18_	[C-M_9_]Y_0.3_(B_0.8_C_0.9_)M_0.9_	9	8.4	—	26.4	11
Fe_48_Cr_15_Mo_14_ B_6_C_15_Dy_2_ = M_77_Dy_2_B_6_C_15_	Fe_0.62_Cr_0.2_Mo_0.18_	[C-M_9_]Dy_0.3_(B_0.8_C_0.9_)M_0.9_	9	8.6	—	26.3	11
Fe_46_Cr_16_Mo_16_B_4_C_18_ = M_78_B_4_C_18_	Fe_0.6_Cr_0.2_Mo_0.2_	[C-M_9_](B_0.5_C_1.3_)M_1.2_	1.2	8.7	—	25.3	81
Fe_44_Cr_16_Mo_16_B_6_C_18_ = M_76_ B_6_C_18_	Fe_0.58_Cr_0.21_Mo_0.21_	[C-M_9_](B_0.8_C_1.3_)M_0.9_	1.2	8.7	—	24.9	81
Fe_42_Cr_16_Mo_16_B_8_C_18_ = M_74_ B_8_C_18_	Fe_0.56_Cr_0.22_Mo_0.22_	[C-M_9_](B_1_C_1.3_)M_0.7_	1.2	8.7	—	24.5	81
Fe_44.3_Cr_5_Mo_12.8_Mn_11.2_Co_5_B_5.9_ C_15.8_ = M_78.3_ B_5.9_ C_15.8_	Fe_0.57_Cr_0.06_Mo_0.16_Mn_0.14_Co_0.06_	[C-M_9_](B_0.8_C_1_)M_1.2_	< 7	8.7	—	24.8	82
Fe_39_Cr_15_Mo_14_Co_9_B_6_C_15_Y_2_ = M_77_Y_2_B_6_C_15_	Fe_0.51_Cr_0.19_Mo_0.18_Co_0.12_	[C-M_9_]Y_0.3_(B_0.8_C_0.9_)M_1_	16	8.5	—	26.3	83
Fe_64_Mo_14_B_6_C_15_Er_1_ = M_78_Er_1_B_6_C_15_	Fe_0.82_Mo_0.18_	[C-M_9_]Er_0.1_(B_0.8_C_0.9_)M_1.1_	3.5	8.8	—	25.7	79
Fe_63.5_Mo_14_B_6_C_15_Er_1.5_ = M_77.5_Er_1.5_ B_6_C_15_	Fe_0.8_Mo_0.18_	[C-M_9_]Er_0.2_(B_0.8_C_0.9_)M_1.1_	3	8.8	—	25.9	79
Fe_63_Mo_14_B_6_C_15_Er_2_ = M_77_Er_2_B_6_C_15_	Fe_0.82_Mo_0.18_	[C-M_9_]Er_0.3_(B_0.8_C_0.9_)M_1_	3	8.8	—	26.2	79
Fe_64_Mo_14_B_6_C_15_Dy_1_ = M_78_Dy_1_B_6_C_15_	Fe_0.82_Mo_0.18_	[C-M_9_]Dy_0.1_(B_0.8_C_0.9_)M_1.1_	3	8.7	—	25.7	79
Fe_63_Mo_14_B_6_C_15_Dy_2_ = M_77_Dy_2_B_6_C_15_	Fe_0.82_Mo_0.18_	[C-M_9_]Dy_0.3_(B_0.8_C_0.9_)M_1.0_	3	8.8	—	26.2	79
Fe_50_Cr_14_Mo_14_B_6_C_14_Y_2_ = M_78_Y_2_B_6_C_14_	Fe_0.64_Cr_0.18_Mo_0.18_	[C-M_9_]Y_0.3_(B_0.8_C_0.8_)M_1.1_	3	8.4	8.43	26.5	84
Fe_50_Cr_14_Mo_14_B_6_C_14_Dy_2_ = M_78_Dy_2_B_6_C_14_	Fe_0.64_Cr_0.18_Mo_0.18_	[C-M_9_]Dy_0.3_(B_0.8_C_0.8_)M_1.1_	3	8.6	8.6	26.7	84
Fe_43.6_Cr_4.9_Mo_12.6_Mn_11.0_Co_4.9_B_5.8_C_15.6_Y _1.5_ = M_77.1_Y_1.5_B_5.8_C_15.6_	Fe_0.57_Cr_0.06_Mo_0.16_Mn_0.14_ Co_0.06_	[C-M_9_]Y_0.2_(B_0.8_C_1_)M_0.9_	>12	8.4	7.8	28.1	82

The critical diameter sizes for fully glassy ingots, estimated and measured densities, and e/u’s are also shown. Calculated mass densities are used only when experimental densities are not available.

The calculation is done by using $\frac{{\rm{e}}}{{\rm{u}}}=\frac{{1.25}^{3}{\rm{\pi }}}{3}\,\times \frac{{\rm{Z}}}{{{\rm{\rho }}}_{{\rm{a}}}.{{\rm{r}}}_{1}^{3}}$, where ρ_a_ is the atomic density (number of atoms per unit volume), *Z* the total number of atoms in the cluster formula, and *r*_1_ the cluster radius of the basic binary clusters^21^. The cluster radius *r*_1_ of a complex alloy is an unknown parameter but can take the value of the relevant basic binary cluster. This is a reasonable simplification because at most M is mainly composed of 3d transition metals of similar atomic sizes like Cr and Mn. Even if large atoms such Mo and Ta are introduced, their amounts are minor so that their influence on *r*_1_ can be ignored. Atomic density ρ_a_ can be transformed from mass density *ρ* by multiplying *ρ* with Avogadro constant *N*_0_ and divided by the average atomic weight Σ*C*_*i*_ *A*_*i*_ (C_i_ and A_i_ are respectively the atomic fraction and atomic weight of element *i* in the alloy). ρ_a_ is also equal to the reciprocal of average atomic volume $\frac{4\pi }{3}\frac{\Sigma {C}_{i}{R}_{i}^{3}}{\eta }$, where *η* is the atomic packing efficiency and is empirically fitted as 0.671 from the experimentally measured densities in Fe-P based metallic glasses following the method reported in ref. 29. Note that here we deal with the global packing efficiency of the whole cluster formula, including both the cluster and the glue atom parts, which is different from the atomic packing efficiency of the cluster itself^39, 85^. By using this atomic packing efficiency, densities can be evaluated for all Fe-P-based compositions as shown in Table 1. e/u = 24.4 for M_75_Mo_4_B_4_C_4_Si_3_P_10_ = [P-M_9_]M_1.3_B_0.5_C_0.5_Si_0.4_P_0.3_ can be calculated using a calculated mass density of 7.2 g/cm^3^.

Typical Fe-metalloid-based metallic glass alloys are collected (mostly BMGs but some ternary alloys with weak glass forming abilities are also included) and explained using the procedures stated above, with their cluster formulas, critical sizes, calculated and experimental densities, and e/u’s illustrated in Table 1. The estimated mass densities *ρ*_cal._ are calculated by using atomic packing efficiency of 0.7 for Fe-B-based alloys, 0.671 for Fe-P-based, and 0.77 for Fe-C-based. It is noted that RE is not commonly used in Fe-B-based BMGs, except one example Fe_71.2_Y_4.8_B_24_ which is understood as a partial substitution of the glue atom Fe by Y. RE elements are practically missing in Fe-P-based BMGs. However, RE’s are often present in Fe-C-based BMGs. Al and Ga, practically missing in Fe-(B, C)-based BMGs, are frequently present in Fe-P-based BMGs, with their amounts taking up to 1 atom in the formula.

**Figure 8:**
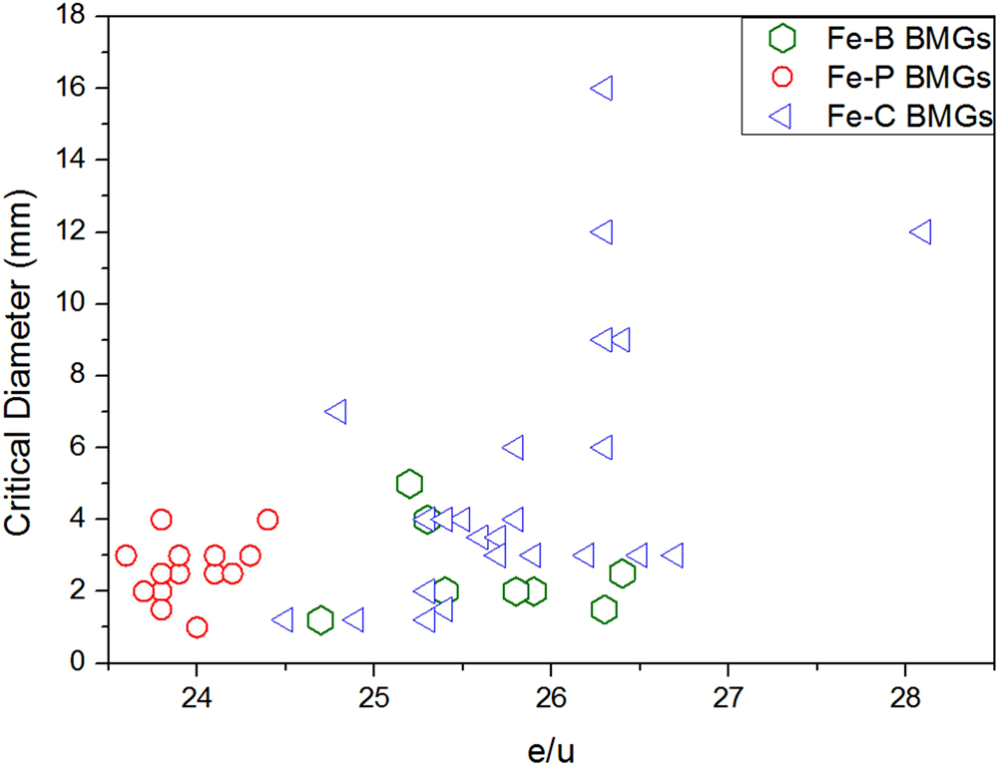
Plot between e/u and critical diameter of Fe-(B,P,C) BMG’s.

As a further exploration of Table 1, the glass forming ability (critical size) is again shown in Fig. 8 as a function of e/u ratio. It is clear that e/u is not well satisfied for Fe-B and Fe-C based BMG’s. This discrepancy can be understood as arising from the uncertain density and cluster radius values. As has been stated, in alloy systems containing covalent bonds, the estimation of atomic densities cannot be made accurate. What is more important is the basic binary cluster radius *r*_1_, which is related to e/u following $\frac{{\rm{e}}}{{\rm{u}}}=\frac{{1.25}^{3}{\rm{\pi }}}{3}\times \frac{{\rm{Z}}}{{\rm{\rho }}a\cdot {{\rm{r}}}_{1}^{3}}$. In general, the cluster radius r_1_ is calculated by averaging all the nearest neighbour distance. However, such an averaging is complicated by multi-alloying, and to simplify the calculation, here we always use those of binary systems and inevitably multiple alloying introduces cluster radius variations. Since e/u is inversely proportional to the cube of *r*_1_, tiny variations induces quite large change in e/u. It is noted that most of the added constituent elements in Fe- (B, C)-based multicomponent BMGs have larger atomic radii, so the alloying should result in increased cluster radii and henceforth decreased e/u values.

For instance, in Fe-C-based BMGs, the composition with maximum glass diameter thickness, Fe_39_Cr_15_Mo_14_Co_9_B_6_C_15_Y_2_, is interpreted by the cluster formula [C-M_9_]Y_0.3_(B_0.8_C_0.9_)M_1_. Here Co, Cr and Mo all show negative enthalpies of mixing with C so that they should prefer the cluster shell sites, just like Fe’s. In Fe-C based binary cluster [C-Fe_9_], 1^st^ neighbour distances are 2.0065 Å (one Fe), 2.0111 Å (one), 2.0197 Å (two), 2.0212 Å (two), 2.3734 (two) and 2.8064 Å (one) and their average value is 2.18362 Å, which is used as the cluster radius *r*_1_. The change in radius Δr as caused by adding other alloying elements is assessed by considering the Goldschmidt radii difference of the new alloying elements with respect to those that they replace in the shell sites (the center atom remains unchanged, being C). The atomic radii of Fe, Co, Cr, and Mo are respectively 1.27, 1.25, 1.28, and 1.40 Å. The cluster radius change can be calculated as the weighted sum of all the atomic radius differences from the alloying elements: Δ*r* = (14/77)*(1.4–1.27) + (15/77)*(1.28–1.27) + (9/77)*(1.25–1.27) ≈ 0.023 Å, where the three radius differences correspond those of Co, Cr, and Mo, respectively. The new cluster radius becomes: r_1_ + Δ*r* = 2.184 + 0.023 = 2.207 Å. With this new radius, e/u is calculated, i.e., 25.5, which is much less than previously calculated using the binary cluster radius.

## Conclusions

The present paper solves an important issue regarding the establishment of the composition formulas for BMGs, via eutectic composition interpretation in Fe-rich metalloid-containing BMGs using the cluster-plus-glue-atom model. The dual-cluster formulas for Fe-rich Fe-(B,P,C) binary eutectics are first proposed: [Fe-Fe_14_]B_2_Fe + [B-B_2_Fe_8_]Fe ≈ Fe_83.3_B_16.7_ for eutectic Fe_83_B_17_, [P-Fe_14_]P + [P-Fe_9_]P_2_Fe ≈ Fe_82.8_P_17.2_ for Fe_83_P_17_, and [C-Fe_6_]Fe_3_ + [C-Fe_9_]C_2_Fe ≈ Fe_82.6_C_17.4_ for Fe_82.7_C_17.3_. The latter formulas in each, being respectively issued from BMG devitrification phases Fe_2_B, Fe_3_P, and Fe_3_C, are then clearly identified, which constitutes a new route towards understanding the composition rule of BMGs. The compositions of existing Fe-based transition metals-metalloid bulk metallic glasses are well interpreted using these basic binary formulas. The 24-electron rule is also verified.
